# From actually toxic to highly specific – novel drugs against poxviruses

**DOI:** 10.1186/1743-422X-4-8

**Published:** 2007-01-15

**Authors:** Katja Sliva, Barbara Schnierle

**Affiliations:** 1 Paul-Ehrlich-Institut, Paul-Ehrlich-Straße 51–59, 63225 Langen, Germany

## Abstract

The potential use of variola virus, the causative agent of smallpox, as a bioweapon and the endemic presence of monkeypox virus in Africa demonstrate the need for better therapies for orthopoxvirus infections. Chemotherapeutic approaches to control viral infections have been less successful than those targeting bacterial infections. While bacteria commonly reproduce themselves outside of cells and have metabolic functions against which antibiotics can be directed, viruses replicate in the host cells using the cells' metabolic pathways. This makes it very difficult to selectively target the virus without damaging the host. Therefore, the development of antiviral drugs against poxviruses has initially focused on unique properties of the viral replication cycle or of viral proteins that can be selectively targeted. However, recent advances in molecular biology have provided insights into host factors that represent novel drug targets. The latest anti-poxvirus drugs are kinase inhibitors, which were originally developed to treat cancer progression but in addition block egress of poxviruses from infected cells. This review will summarize the current understanding of anti-poxvirus drugs and will give an overview of the development of the latest second generation poxvirus drugs.

## Background

The worldwide eradication of the naturally occurring smallpox virus, variola, in 1980 resulted in a decreased demand for the development of therapies [1]. Due to recent worldwide political developments, variola is nowadays widely regarded as one of the most significant bioterrorist threats, reestablishing the need for efficient therapy for poxvirus infection [2,3]. The impact of a smallpox virus attack in the human population today would be even more catastrophic than during the last century, since the vaccination programs were suspended worldwide around 1976 [4]. The lethality of the disease (up to 40%) and its ease of transmissibility have prompted the CDC (Center for Disease Control and Prevention), an agency recognized as the leading United States government agency for protecting public health and safety, to place variola virus at the top of the high-threat (Category A) agents list [5]. In addition to the bioweapon threat, there is a natural public threat arising from monkeypox virus, a virus that produces a disease in man that closely resembles smallpox. Monkeypox exists naturally in western and central Africa, but 72 cases were also reported in the United States in 2003 [2,6,7].

Variola and monkeypox viruses belong to the family of poxviridae, which consists of a collection of large, enveloped, double-stranded DNA viruses that are distinguishable by their unique morphology and cytoplasmic site of replication [8]. Poxviruses infect most vertebrates and invertebrates, causing a variety of diseases of veterinary and human medical importance. The poxvirus family is divided into two main subfamilies, the *chordopoxvirinae*, which infect vertebrates, and the *entomopoxvirinae*, which infect insects. *Chordopoxvirinae *are further divided into eight genera. One of these is orthopoxvirus, which includes the human pathogens variola virus and monkeypox virus, and others which infect humans, including cowpox and vaccinia virus (VACV). There are at least two natural strains of variola virus: *variola major *with a case fatality rate of 30–40% and *variola minor*, with a much reduced fatality rate of approximately 1%.

Poxviruses enter the oropharyngeal and respiratory mucosa, and proliferate in the regional lymph nodes, multiplying in particular in the reticulo-endothelial system. However, the cellular entry mechanism is unknown in terms of fusion proteins and cell receptors [9,10].

**Figure 1:**
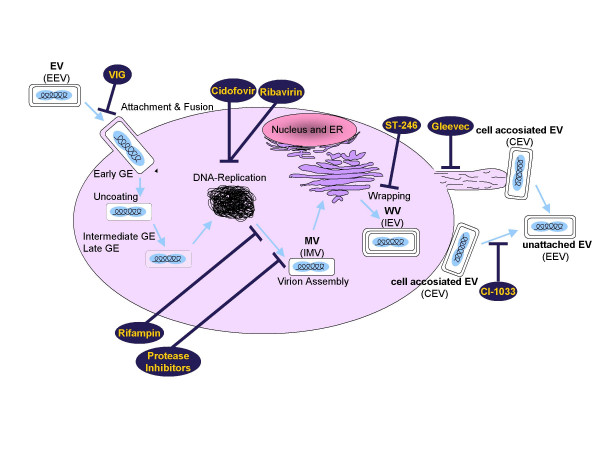
Schematic overview of the VACV replication cycle, the different virion forms and the point of action of anti-poxvirus drugs.

The 191 kbp VACV DNA genome encodes at least 263 gene products. Their expression is regulated in a temporal fashion during the viral replication cycle, which begins with entry of the virus into the host cell and terminates with the assembly of complex macromolecular structures to form an infectious particle [11]. Although the molecular details of poxvirus assembly and differentiation remain controversial, the most widely accepted scenario involves the generation of at least three forms of infectious particles (Figure 1). The nomenclature used in this review follows a recent proposal by Moss [9]. The multiple infectious forms differ from one another by their outer membrane. Directly after the attachment and fusion of the virus with the host cell, the virus is uncoated and the early gene expression is initiated. At this point, the DNA replication occurs and is followed by intermediate and late gene expression. After viral DNA replication, progeny DNA molecules, virion enzymes and structural proteins assemble to form the pre-virion particles now referred to as mature virion (MV). MVs are the simplest and most abundant form and have no additional membranes and have previously been called the intracellular mature virion (IMV). MVs then acquire membranes, whether this is one or two membranes remains controversial; however, the current perception prefers the single membrane model [9]. A portion of the MV then become enveloped with additional membranes derived from the trans-Golgi apparatus [12,13] or endosomal cisterna [14]. This MV form, which is surrounded by two membranes, is referred to as a wrapped virion (WV) [9] instead of the previous intracellular enveloped virion (IEV), as the MV is already enveloped. Following migration to the cell surface, the outer WV membrane fuses with the plasma membrane resulting in exocytosis, which gives rise to extracellular enveloped virus (EV) [15]. The EV can either remain associated with the cell (formerly CEV = cell-associated virus) or become unattached and released as extracellular enveloped virus (EEV) [16]. The associated form is usually predominant and primarily responsible for cell-to-cell spread via actin tails [17,18]. Figure 1 shows a schematic view of the VACV replication cycle and the different virion forms.

Among the approximately 200 genes encoded by variola virus are many that code for gene products that are needed for cytoplasmic transcription and replication of the virus. Therefore, strategies which block such key enzymes in the replication and maturation of poxviruses provide potential targets for therapeutic intervention.

### Animal models

Animal models that are predictive for human disease outcome are an important component of drug development. There are a number of models in use, but none of them captures all aspects of the human orthopoxvirus infections. The most relevant are models in which poxvirus replication is initiated in the periphery and followed by systemic virus spread, as occurs during variola virus infections in humans.

A frequently used, straightforward model is the intranasal VACV infection of mice. It results in local replication and systemic disease; however, a large inoculation dose needs to be applied [19]. Ectromelia virus infection of mice strongly resembles the pathogenicity of human smallpox, although the time course of infection and disease progression is much shorter [20]. The parameter that is monitored in both models is disease progression and the primary end-point is mortality.

Monkeypox virus infections of nonhuman primates have also been used to evaluate the efficacy of smallpox vaccines. A major drawback of these models is the intravenous administration, which bypasses the mucosa and produces a lesional disease characteristic of post secondary viremia. However, disease progression can be monitored more precisely and virus yields can be measured in serum and by quantifying lesion numbers [21].

### Prevention of clinical symptoms: First therapeutic approaches towards a treatment of poxvirus infection

From about 2000 BC onwards, treatment of smallpox infections basically involved prevention of clinical symptoms, as no curative therapy was available. Early attempts to control smallpox began in ancient Egypt through intranasal insufflations of dried crusts of smallpox lesions. In 18^th ^century Europe, the method consisted of subcutaneous injection of fluid from smallpox pustules or scabs. This practice, known as variolization, caused severe cases of smallpox in about 1 in 200 inoculations. However the fatality rate in variolated individuals was considerably lower than in those who got naturally infected.

The breakthrough in prevention began in the 18^th ^century, when doctors observed that milkmaids who had contracted cowpox were resistant to infection with smallpox. Edward Jenner reasoned and finally showed in 1796 that infectious material from cowpox lesions provided protection from smallpox. The term vaccination was coined in 1803 from the Latin word for cow (*vacca*) [22]. Based on these findings, the World Health Organization undertook a global smallpox vaccination program in 1967. At that time, 10 to 15 million cases of the disease occurred each year with more than 2 million deaths. VACV, a related but relatively nonpathogenic poxvirus was administered throughout the world, and this practice resulted in the eradication of smallpox [1]. When the vaccine, live VACV, is inoculated into the superficial layers of the skin (skin scarification), the virus grows locally and induces an immune reaction that protects against smallpox [23]. This program of intensively vaccinating all humans in a ring surrounding every suspected case of variola infection was successful in part because smallpox is a human-only disease. There are no animal reservoirs to reintroduce the virus into the human population. The last occurrence of endemic smallpox was in Somalia in 1977, and the last human cases were laboratory-acquired infections in 1978 [24].

### Treatment of acute infections

Vaccination programs were suspended worldwide in 1976 and the human population now lacks immunity against poxviruses, making them attractive as bioweapons. Complications associated with VACV vaccination, which might occur in immune-compromised individuals, also increase the demand for drugs that can be used to treat acute infections. The principles of treatment can be summarized as follows: Passive immunization, antiviral small molecule drugs that were identified by random screens and inhibit viral replication and drugs that target systemic spread of poxviruses.

#### Passive Immunization

Passive immunization is one method to treat variola-infected individuals. In this procedure, antibodies obtained from the plasma of repeatedly actively vaccinated individuals are infused into infected individuals or those with a high risk of becoming infected. The antibodies provide protection against disease for two to three weeks, as this is the time they remain active in the body. Although short-lived, passive immunization provides immediate protection, unlike active immunization, which can take weeks to develop. Consequently, passive immunization can be lifesaving when a person has been infected [25].

The development of humanized monoclonal antibodies against neutralizing epitopes that are conserved between monkeypox and variola viruses are therefore promising advanced tools for passive immunization against poxviruses. However, a safe, orally active antiviral drug for the prevention or treatment of smallpox infection is needed to provide an alternative to the elaborate antibody-based passive immunization. It has been shown recently that antiviral treatment is more effective than smallpox vaccination upon lethal intratracheal infection of cynomolgus monkeys (*Macaca fascilaris*) with monkeypox virus [26]. Today, there are no FDA-approved drugs available for the treatment of smallpox; however, there are drugs in early preclinical development and approved drugs with off-target effects. All drugs discussed below, and their point of attack in the viral lifecycle, are summarized in Figure 1.

#### Small molecule drugs that inhibit viral replication

The search for antiviral agents acting against poxviruses, without knowing their mode of action, started when the thiosemicarbazones, introduced in 1946 by Domagk et al. [27] as tuberculostatic (antituberculous) agents, were also found to be active against VACV [28]. Hamre et al. found that the drug was able to reduce infectivity in eggs and mice by up to 70%. The observation that benzaldehyde thiosemicarbazone did not inactivate vaccinina virus *in vitro *proved that the antiviral effect of this drug was intracellular. This important work was continued by Bauer [29] and culminated in the demonstration by Bauer et al. [30] in 1963 that the thiosemicarbazone derivative methiazone (Marboran, N-methylisatin 3-thiosemicarbazone) was effective in the prophylaxis of smallpox. Methiazone was first applied in 1962 to a smallpox-infected patient who suffered from infantile eczema and did not respond to treatment with anti-vaccinia gamma globulin [31]. The boy showed rapid recovery after treatment and was the first illustration of the use of an antiviral drug in man [32]. From then on trials with methiazone showed that the drug not only had an antiviral effect in infected patients but was also effective as a prophylaxis of smallpox. Promising results have also been reported in the treatment of complications of smallpox vaccination. The principal side effect of severe vomiting was considered a justifiable risk at the time [33], but is unacceptable for today's use.

Another approved drug is rifampin, which is still one of the most effective tuberculostatic drugs today. It inhibits the growth of poxviruses by preventing the cleavage of precursor proteins and is effective against poxviruses in high doses (100 μg/ml), which makes its clinical use rather unlikely [34].

For more than 25 years, VACV has been included in the panel of viruses that are evaluated for their susceptibility to a large variety of different classes of compounds. This search has yielded a wealth of substances, lead compounds and approaches that have proved effective against VACV [35]. Most of the compounds that have been identified as anti-VACV agents are nucleoside analogs that fall into different categories [36]. The above-mentioned methiazone belongs to the thiosemicarbazones. Ribavirin, an antiviral drug which is active against a number of DNA and RNA viruses including VACV, belongs to the IMP dehydrogenase inhibitors. There are numerous other nucleoside or nucleotide analogs that have been reported to have antiviral activity against VACV *in vitro*, such as SAH hydrolase (S-adenosylhomocysteine hydrolase) inhibitors, orotidine-5'-monophosphate (OMP) decarboxylase/cytidine triphosphate (CTP) synthetase inhibitors and thydidylate synthase inhibitors. All these compounds inhibit VACV replication *in vitro *and some of the SAH hydrolase inhibitors suppress the consequences of a VACV infection *in vivo *[37].

The only antiviral agent currently approved for use against orthopoxviruses is cidofovir (CDV-Vistide), which belongs to the acyclic nucleoside phosphonates. Cidofovir is currently licensed for the treatment of cytomegalovirus (CMV) retinitis and is thought to act by inhibiting the cytomegalovirus DNA polymerase, a target shared with the poxviruses. It has demonstrated antiviral activity against poxviruses *in vitro*, and against cowpox and vaccinia viruses in mice [38,19,39]. It is also thought to inhibit the activity of the proofreading exonuclease, leading to error-prone DNA synthesis during poxvirus replication [4]. However, its use for the treatment of VACV adverse reactions is restricted under an investigational new drug (IND) protocol. Under the IND, cidofovir can only be used when VIG (vaccinia immune globulin) is not efficacious. Renal toxicity is a known adverse reaction of this drug. In recent years a bioavailable variant of orally available cidofovir has been developed: HDP-cidofovir (hexadecyloxypropyl-CDV) [40,41]. This modification (adding a lipid) resulted in a new drug, which showed a 100-fold enhanced potency at blocking smallpox virus reproduction in tissue-culture cells and might prove more clinically relevant in the future [42,43].

Another nucleotide analog is adefovir [44]. However, although adefovir shows significant *in vitro *antiviral activity against poxviruses, its efficacy as a therapeutic agent for smallpox is currently uncertain. In addition to its activity against VACV replication in tissue culture, it has a good oral availability and has been approved for treatment of Hepatitis B [45], making it a serious candidate. This compound needs to be evaluated further for its activity against monkeypox and variola viruses before its real potential is known [46].

#### Drugs that target systemic spread

In the last two years, a novel class of anti-poxvirus drugs has emerged. These compounds do not inhibit viral replication directly but rather reduce virus spread. They decrease the release of EEV from cells, which is considered to be the main mechanism for rapid spread of poxviruses in the infected host. EEV are actively extruded from cells by interaction with actin tails. Actin tail formation is regulated by phosphorylation of the viral protein A36 by the cellular tyrosine kinase c-Src, which can also be activated by the epidermal growth factor receptor (EGFR), a membrane-anchored receptor tyrosine kinase. A36R is located in the membrane surrounding the EEV and is required for actin polymerization and virulence [47,48]. Therefore, specific kinase inhibitors for either kinase are able to inhibit spread of poxviruses and greatly increase animal survival after VACV infection.

One of these drugs is Gleevec (also called STI-571, imatinib mesylate or Glivec), which was licensed for use in chronic myeloid leukemia and is approved for use in humans. Gleevec inhibits Abl-family tyrosine kinases and, to some extent, EGFR, and has been shown to block the egress of VACV from infected cells [49,50]. Gleevec is not the first example of poxvirus inhibition by a kinase inhibitor, but it is the first kinase-targeted drug approved for use in humans that exhibits antiviral properties. The Erk inhibitor U1026 blocks VACV replication in cultured cells [51] and CI-1033, an EGFR kinase inhibitor, has similar effects to Gleevec and is also able to rescue mice from a lethal intranasal VACV challenge infection [50]. Poxviruses encode a growth factor (VGF) which has high homology to EGF. These EGF-like growth factors are carried by poxviruses to facilitate viral pathogenesis. VGF binds to the EGFR and the effect of Cl-1033 is most likely due to reduced EEV release which is normally triggered by VGF. Two EGFR kinase inhibitors have obtained FDA approval (gefitinib and erlotinib) and both drugs might have anti-poxvirus properties.

A different type of drug is ST-246, which was identified by high-throughput screening of a small-molecule compound library for inhibitors of orthopoxvirus replication. Its target is the F13L gene product, which is required for production of extracellular virus. ST-246 also reduces extracellular virus formation and, although it has little effect on the production of intracellular virus, it can protect mice from lethal orthopoxvirus infection [50].

Further understanding of viral egress might identify novel targets for intervention. The use of host cellular signaling pathway blockades as antiviral chemotherapy has the advantage that drug resistance cannot readily develop and is highly promising. However, cellular targets always require the consideration of unwanted side effects, which have been described recently for kinase inhibitors [52].

### Potential new drug targets

A large variety of gene products are essential or have exactly defined functions in the viral replication cycle. This knowledge can be exploited for the precise design of drugs targeting these proteins [46]. Proteins with enzymatic activity, such as kinases or phosphatases, are especially good drug targets, because inhibitors might be easily identified by high-throughput screens.

For example, the H6R gene encodes the viral topoisomerase 1B (vTOPO), which is essential for DNA processing. It shares extensive structural and mechanistic features with the human type 1B enzyme (hTOPO). However, despite these similarities, there are sufficient differences to allow selective targeting of the viral variant. Two coumarin drugs (novobiocin and coumermycin) are potent inhibitors of vTOPO, which show little effect on hTOPO [53]. These early findings indicate that it is possible to discover compounds that interact selectively with these enzymes. Bond et al. [54] performed a high-throughput screen which resulted in the identification of a different class of small-molecule inhibitors that potently inhibit DNA supercoil relaxation by vTOPO, and obtained promising results for the therapeutic use of these compounds.

In addition, there are some protein kinases with defined biochemical activity but unclear functions in viral replication. Kinases are the most frequently used drug targets, and inhibitors with high specificity have been identified for a multitude of cellular kinases [55]. Variola and vaccinia viruses encode two essential protein kinases with well characterized kinase activity [56,57] – the VACV protein kinases 1 and 2 (VPK1 and VPK2). VPK1 is the B1R kinase [56]. The H5R gene product in VACV is a natural substrate of this enzyme [58], and both the H5 and B1 proteins are present in virions [58]. The B1 protein seems to be involved in viral DNA synthesis ([59], as mutants with temperature sensitive (ts) defects in B1 fail to synthesize viral DNA [57], but it is unclear how this protein functions in this process or in phosphorylation of H5R. A specific inhibitor of B1R might be developed into an antiviral drug with an adequate safety profile.

The second protein kinase of interest, the VPK2, is the F10L protein kinase. F10L is required for viral replication and is the major kinase encapsidated in virions. The product of this gene shows autophosphorylation activity [60], but also appears to be involved in the phosphorylation of the A17 protein, which is one of its natural substrates. Mutant viruses with ts lesions in F10 appear to be arrested at a very early stage in virion morphogenesis [61,62]. Both kinases could be models for the design of specific inhibitors as antiviral drugs with a clearly defined effect spectrum.

Other possible starting points for drug design are nucleoside and nucleotide kinases like, for example, J2R and A48R. The thymidine kinase J2R and thymidylate kinase A48R are not required for viral replication, but J2R is thought to have an attenuating influence on viral replication *in vivo *[63]. A number of drugs that have been approved for the treatment of herpes virus infections are active against VACV *in vitro*. Other compounds that have been tested in phase II/III clinical trials for these infections are also active against orthopoxviruses [64]. As all these active compounds are closely related halogenated uracil analogs or phosphate nucleotides, the current hypothesis is that the effect of these nucleoside analogs on orthopoxviruses is not limited by their ability to inhibit the VACV DNA polymerase but by their ability to be phosphorylated by the thymidine kinase J2R. Clearly, more research into the substrate specificity of J2R is required to open the possibilities for finding highly active and non-toxic nucleoside analogs [46].

Another interesting candidate for drug target design could be the protein phosphatase encoded by the H1L. H1L has been shown to be essential for viral replication, as it has not been possible to generate recombinant viruses with deletions in this gene and recombinant viruses with repressible expression of H1L show reduced replication [65]. Designing drugs which target this essential protein could stop viral spread throughout infected people.

The host range and pathogenicity of poxviruses result from the interactions of virally encoded factors with the host immune system. The limited host range of variola virus is thought to relate largely to the unique association of viral gene products with the countless host signaling pathways. Interference with these factors might be a novel approach to develop drugs that reduce or ablate variola virus pathogenicity.

## Conclusion

Currently there are no FDA approved drugs on the market for the treatment of poxvirus infections, however kinases inhibitors show very good anti-poxvirus activity as off-target effect and have the potential for clinical applications. Essential viral gene products in the poxviral replication cycle have been identified that are potential targets for new drug development, but also more basic research is required to identify essential viral enzymes or pathogenicity factors, which will enable the development of more effective and specific anti-poxvirus drugs. Hopefully, these drugs will make poxviruses unattractive as bioweapons.

## Competing interests

The authors declare that they have no competing interests.

## Authors' contributions

KS and BS contributed equally to conception, design and acquisition of data. Both have been involved in drafting the manuscript and have given final approval of the version to be published.
